# FBXW7 in breast cancer: mechanism of action and therapeutic potential

**DOI:** 10.1186/s13046-023-02767-1

**Published:** 2023-09-02

**Authors:** Siyu Chen, Ping Leng, Jinlin Guo, Hao Zhou

**Affiliations:** Chongqing Key Laboratory of Sichuan-Chongqing Co-Construction for Diagnosisand, Treatment of Infectious Diseases Integrated Traditional Chinese and Western Medicine, College of Medical Technology , Chengdu University of Traditional Chinese Medicine, Chengdu, China

**Keywords:** FBXW7, Breast cancer, ncRNAs regulation, Angiogenesis, Immune escape, Drug resistance, Therapeutic strategies

## Abstract

Breast cancer is one of the frequent tumors that seriously endanger the physical and mental well-being in women. F-box and WD repeat domain-containing 7 (FBXW7) is a neoplastic repressor. Serving as a substrate recognition element for ubiquitin ligase, FBXW7 participates in the ubiquitin–proteasome system and is typically in charge of the ubiquitination and destruction of crucial oncogenic proteins, further performing a paramount role in cell differentiation, apoptosis and metabolic processes. Low levels of FBXW7 cause abnormal stability of pertinent substrates, mutations and/or deletions in the FBXW7 gene have been reported to correlate with breast cancer malignant progression and chemoresistance. Given the lack of an effective solution to breast cancer's clinical drug resistance dilemma, elucidating FBXW7's mechanism of action could provide a theoretical basis for targeted drug exploration. Therefore, in this review, we focused on FBXW7's role in a range of breast cancer malignant behaviors and summarized the pertinent cellular targets, signaling pathways, as well as the mechanisms regulating FBXW7 expression. We also proposed novel perspectives for the exploitation of alternative therapies and specific tumor markers for breast cancer by therapeutic strategies aiming at FBXW7.

## Introduction

Breast cancer is the most prominent type of cancer in females globally, with the second highest fatality rate [1]. Chemotherapy remains the backbone of therapy for advanced breast cancer because advanced patients make up most breast cancer patients, but this comes with side effects such as drug resistance and organ damage, as well as a higher risk of metastasis and recurrence [2]. It is apparent that metastasis and chemotherapy resistance has substantially harmed the survival of patients with breast cancer, and better-targeted therapeutic options are required to arrest its progression.

**Fig. 1:**
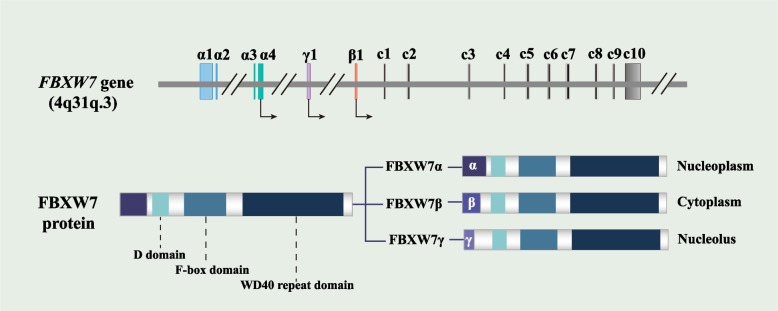
Structure of the FBXW7 gene situated on human chromosome 4q31q.3 and protein isoforms of FBXW7. All FBXW7 isoforms have a locus structure with 10 exons (c1-c10) and 4 introns. Additionally, While FBXW7β and FBXW7γ are both encoded by a single exon (β1 and γ1), FBXW7α has four distinct exons (α1, α2, α3, and α4). Apart from the NH2 terminal section, all three isomers of FBXW7 share the D domain, the F-box domain, and the WD40 repeat domain. FBXW7α, FBXW7β, and FBXW7γ are present in the nucleoplasm, cytoplasm, and nucleolus, respectively. FBXW7, F-box and WD repeat domain-containing 7

**Fig. 2:**
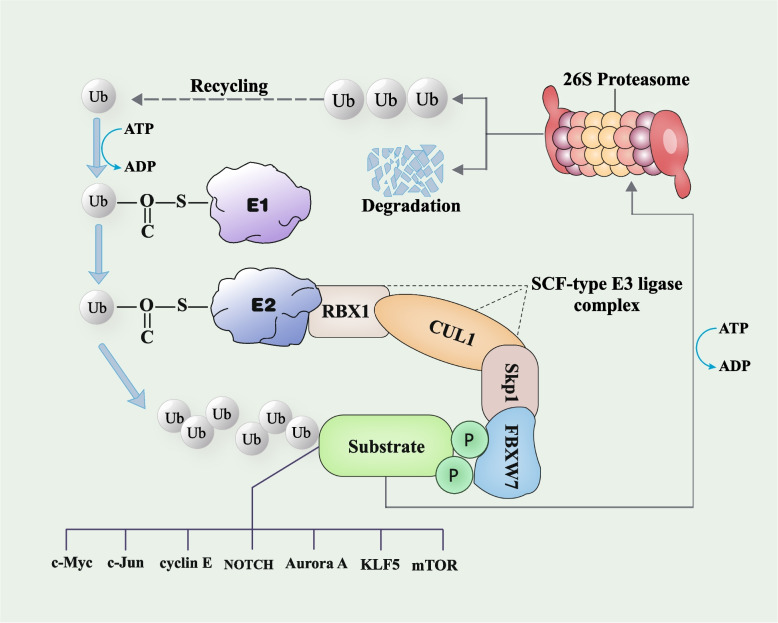
SCF^FBXW7^-mediated ubiquitination and degradation of substrate proteins. Ubiquitin molecules and phosphorylation are denoted by Ub and P, respectively. Before being transported to E2, the ubiquitin molecule is first activated by E1 via a thioester bond with associated ATP hydrolysis. The SCF-type E3 ligase then identifies the phosphorylated substrate through the substrate receptor FBXW7 and adds ubiquitin from E2 to the substrate. Once enough ubiquitin chains have been attached to the substrate, they are transferred in an ATP-dependent manner to the 26S proteasome for hydrolysis. While the ubiquitin molecule circulates, the substrate protein is broken down into a brief peptide. FBXW7 substrates mainly include c-Myc, c-Jun, cyclin E, NOTCH, Aurora A, KLF5, and mTOR. FBXW7, F-box and WD repeat domain-containing 7. E1, ubiquitin-activating enzymes. E2, ubiquitin-conjugating enzymes. E3, ubiquitin ligase. SCF, Skp1-Cullin1-F-box. RBX1, RING-finger protein 1. CUL1, Cullin1. Skp1, S-phase kinase-associated protein 1. KLF5, Kruppel-like factor 5. mTOR, mechanistic target of rapamycin

The Skp1-Cullin1-F-box (SCF) complex's substrate receptor, F-box and WD repeat domain-containing 7 (FBXW7), is a member of the FBXW family of F-box proteins [3]. 13 encoding exons and 4 untranslated introns make up the roughly 210 kb-long human FBXW7 gene, located at 4q31q.3 chromosome, which is absent in ~ 30% cancers [4]. There are 3 different FBXW7 isoforms: FBXW7α, FBXW7β, and FBXW7γ, with distinct exons created via selective splicing. The isoforms have different cellular localizations. The nucleoplasm, cytoplasm, and nucleolus are the respective locations of FBXW7α, FBXW7β, and FBXW7γ [5] (Fig. 1). The following functionally significant domains are found in the regions shared by all FBXW7 subtypes: the D domain, which promotes the formation of FBXW7 dimers; the WD40 domain, which recognizes substrates; and the F-box domain, which connects with the SCF group subunits RING-finger protein 1 (RBX1), Cullin1 (CUL1), and S-phase kinase-associated protein 1 (Skp1). The SCF complex acts as a ubiquitin ligase (E3) to ubiquitinate proteins and initiate proteasomal degradation along with ubiquitin-activating enzymes (E1) and ubiquitin-conjugating enzymes (E2) [6]. FBXW7 may recognize substrates that have been phosphorylated at specific residues through the conserved Cdc4 phospho-degron (CPD) motif. FBXW7 employs the phosphorylation of substrates, which is mediated mostly by Glycogen Synthase Kinase 3 beta (GSK3β), to encourage substrate recruitment, ubiquitination, and proteasomal degradation [7]. By encouraging the ubiquitination and destruction of associated oncogenic proteases such c-Myc, Kruppel-like factor 5 (KLF5), c-Jun, NOTCH, cyclin E, mechanistic target of rapamycin (mTOR), and Aurora A, FBXW7 inhibited the formation of tumors [8–14] (Fig. 2). Recent evidence has connected abnormal FBXW7 expression to breast cancer growth, metastasis, and drug resistance [15–17]. Research in mouse models confirmed that FBXW7 deletion causes mammary epithelial cell degeneration with invasive cancer transformation and that the number of transplanted tumor nodules in the lungs of FBXW7 gene-deficient mice is significantly increased [18, 19]. Additionally, inactivating FBXW7 leads to a drop in double-stranded RNA (dsRNA) in mice tumor cells, triggering an altered immune microenvironment and anti-PD-1 resistance [20]. Breast cancer development is affected by associated regulators, FBXW7, and protein substrates crosstalk at the molecular level. Therefore, to break the clinical resistance bottleneck and achieve an early targeted diagnosis, it is necessary to elucidate FBXW7's biological functions in breast cancer. In this review, we highlight the functional role of FBXW7 in the spectrum of malignant behaviors in breast cancer, ubiquitination targets, and the mechanisms of FBXW7 expression. Furthermore, we also summarize breast cancer therapeutic strategies targeting FBXW7, providing novel perspectives for the exploration and innovation of clinically targeted agents and specific diagnostic markers.

## The Regulation of FBXW7 Expression

### FBXW7 regulation at transcriptional level

**Fig. 3:**
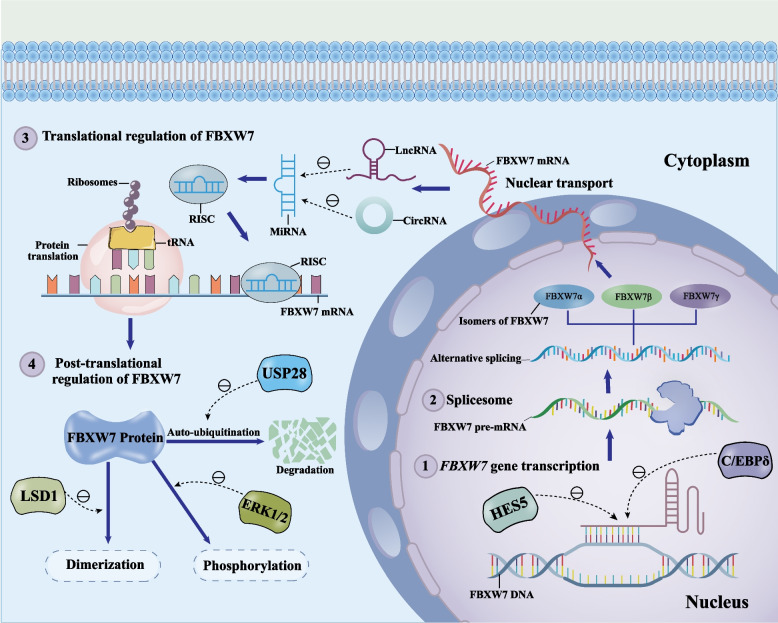
Mechanisms of intracellular FBXW7 regulation at the transcriptional, translational, and post-translational levels. (1) At the transcriptional level, C/EBPδ and HES5 inhibit the FBXW7 gene’s transcription. (2) During FBXW7 pre-mRNA splicing, three isoforms α, β, and γ are produced through alternative splicing. (3) At the translational level, ncRNAs control the expression levels of the FBXW7 mRNA and protein. MiRNAs first form RISC and then bind to the 3' UTR of FBXW7 and regulate its expression, while lncRNAs and circRNAs restore the expression levels of FBXW7 mRNA and protein by sponge-attracting miRNAs. (4) At the post-translational level, multiple upstream factors regulate FBXW7 dimerization, phosphorylation, and auto-ubiquitination. LSD1 inhibits FBXW7 dimerization, ERK1/2 promotes FBXW7 phosphorylation, and USP28 inhibits FBXW7 auto- and substrate ubiquitination. FBXW7, F-box and WD repeat domain-containing 7. HES5, HES family bHLH transcription factor 5. C/EBPδ, CCAAT/enhancer-binding protein-delta. LncRNA, long non-coding RNA. CircRNA, circular RNA. MiRNA, microRNA. RISC, RNA-induced silencing complex, USP28, ubiquitin-specific proteases 28. LSD1, lysine-specific demethylase 1. ERK1/2, extracellular signal-regulated kinases 1 and 2

Given the identification of FBXW7 mutations and/or deletion in a range of malignancies, abnormal regulation of FBXW7 expression may be a trigger for carcinogenesis. FBXW7 has multiple transcriptional regulators, including the tumor suppressor p53, the transcriptional repressors RAN binding protein 10 (RANBP10) and HES family bHLH transcription factor 5 (HES5), the transcriptional activator CCAAT/enhancer-binding protein-delta (C/EBPδ) and 27-hydroxycholesterol (27-HC) [21–25] (Fig. 3). A p53 binding site (p53BS) has been reported on exon 1b of the FBXW7 gene. p53 activates FBXW7 transcription through p53BS, whereas p53 loss in breast cancer inhibits FBXW7 expression, increasing NOTCH5 receptor activation and chemotherapy resistance [26, 27]. 27-HC is an oxysterol that activates estrogen receptor-positive (ER +) breast cancer cell growth by stimulating ERα [28]. The transcriptional activity of FBXW7 is repressed by 27-HC, hence attenuating c-Myc turnover and promoting MCF-7 cells proliferation [25]. The leucine zipper protein family member C/EBPδ, generally stimulates substrate transcription and slows tumor growth. However, C/EBPδ appears to exert an opposite modulating effect on FBXW7 [29]. Most of these transcriptional elements negatively regulate the mRNA levels of FBXW7.

### Non-coding RNAs regulate FBXW7 protein

#### MicroRNAs

MicroRNAs bind to the 3' untranslated region (3' UTR) of the mRNA to control FBXW7 protein translation and mRNA degradation (Fig. 3). Chen et al*.* revealed that miR-194 suppresses protein translation by specifically combining with the 3' UTR of FBXW7, leading to a rise in the synthesis of cyclin D and cyclin E and the promotion of breast cancer cell proliferation [30]. Additionally, microRNAs' control over FBXW7 has a role in breast cancer metastasis caused by the epithelial-mesenchymal transition (EMT). Jiang et al*.* revealed that miR-27a, an upriver controller gene of FBXW7, restricts its expression at the protein expression level, resulting in EMT occurrence and breast cancer metastasis [31]. Related studies on FBXW7 regulation by miRNAs are summarized in Table 1.

**Table 1:** Non-coding RNAs Regulation of FBXW7 at Translational Level

ncRNAs	Types of ncRNAs	Cancer types	Expression in cancer	Regulation of FBXW7	Target factor	Ref
MiR-194	MicroRNA	BC	Upregulation	Downregulation	↑Cyclin D; ↑Cyclin E	[30]
MiR-27a	MicroRNA	BC	Upregulation	Downregulation	↑EMT	[31]
MiR-182-5p	MicroRNA	TNBC	Upregulation	Downregulation	↑TLR4; ↑NF-κB	[32]
MiR-223-3p	MicroRNA	BC	Upregulation	Downregulation	↑EMT	[33]
MiR-182	MicroRNA	BC	Upregulation	Downregulation	↑HIF-1α; ↑VEGF-A	[34]
MiR-188-5p	MicroRNA	BC	Upregulation	Downregulation	↑c-Myc	[35]
MiR-32	MicroRNA	BC	Upregulation	Downregulation	Not applicable	[36]
CircFBXW7	CircRNA	TNBC	Downregulation	Downregulation	↑MiR-197-3p	[37]

↑, upregulation in cancer tissues. ↓, downregulation in cancer tissues. *FBXW7* F-box and WD repeat domain-containing 7, HIF-1α Hypoxia inducible factor 1 alpha, *VEGF-A* Vascular endothelial growth factor A, *TLR4* Toll-like receptor 4, *NF-κB* Nuclear factor-kappa B, *EMT* Epithelial-mesenchymal transition. *BC* Breast cancer. *TNBC* Triple negative breast cancer

#### CircRNAs

A unique family of single-stranded non-coding RNA molecules called circular RNAs (circRNAs) lacks polyadenylate tails, 5′ to 3′ polarities, and have closed-loop topologies [38]. Akin to long non-coding RNAs (lncRNAs), the majority of circRNAs serve as competing endogenous RNAs (ceRNAs) for miRNAs that modulate FBXW7 (Fig. 3). Liu et al*.* demonstrated that hsa_circ_0022742 reverses the level of repressed FBXW7 expression by sponging miR-503-5p [39]. CircRNAs are typically regarded as incapable of encoding proteins. Recently, however, Yang et al*.* uncovered that circFBXW7 can bind FBXW7α competitively with ubiquitin-specific proteases 28 (USP28) by encoding the FBXW7-185aa protein, thereby inhibiting the deubiquitination of FBXW7α by USP28 [40]. Moreover, Ye et al*.* demonstrated that circFBXW7 may destabilize c-Myc by competing for sponging miR-197-3p, boosting the protein level of FBXW7, in addition to encoding protein. This prevents the development and migration of triple-negative breast cancer (TNBC) cells [37]. Related studies on FBXW7 regulation by circRNAs are summarized in Table 1.

### FBXW7 regulation at post-translational level

The regulation of post-translational modifications of FBXW7 consists of several key processes, including dimerization, phosphorylation, auto-ubiquitination, and deubiquitination.

#### Dimerization

In essence, FBXW7 dimerization is a superhelical structure formed by D domain precursors with spatial variability that enhances the FBXW7's capacity to bind to a diversity of inferior degraders and encourages the ubiquitination and destruction of substrates [41, 42]. Notably, some oncogenic factors could promote cancer development by regulating the dimerization of FBXW7. For example, lysine-specific demethylase 1 (LSD1) blocks FBXW7 dimerization by acting as a pseudosubstrate rather than a demethylase to bind to FBXW7, leading to a reduction in oncoprotein turnover by promoting FBXW7 autoubiquitination [43] (Fig. 3). Despite the current lack of clarity regarding the role played by FBXW7 dimers in breast cancer progression, making clear the crosstalk between dimerization in FBXW7 and substrate ubiquitination could provide a potential molecular mechanism for cellular regulation.

#### Phosphorylation

Post-translational phosphorylation of FBXW7 is catalyzed by multiple kinases, including extracellular signal-regulated kinases 1 and 2 (ERK1/2), polo-like kinase 1 and 2 (PLK1/2), phosphoinositide 3-kinase (PI3K), and protein kinase C (PKC) [44–47] (Fig. 3). It was reported that the regulation of FBXW7 phosphorylation by associated enzymes has facilitated the turnover of FBXW7 itself [44]. Neuregulin-1 (NRG1)-activated ERK1/2 stabilizes downstream c-Myc recruitment to the FOS-like 1 and AP-1 transcription factor subunit (Fra-1) promoters by promoting FBXW7 phosphorylation, in turn facilitating TNBC metastasis [47]. Meanwhile, phosphorylation of FBXW7 by PI3K at S227 inhibits the self-ubiquitination, which consequently promotes the turnover of downstream targets c-Myc and cyclin E [46].

#### Auto-ubiquitination and deubiquitination

At the post-translational level, auto-ubiquitination and deubiquitinating enzymes (DUBs) modulate the degradation of the FBXW7 protein. According to reports, F-box proteins catalyze auto-ubiquitination in the SCF complex in a manner that is SCF-dependent [48]. Chen et al*.* displayed that COP9 signalosome 6 (CSN6) promotes auto-ubiquitination of FBXW7, causing an increase in c-Myc expression [49]. Moreover, FBXW7 dimerization also facilitates its auto-ubiquitination [42]. Intriguingly, the regulation of FBXW7 by USP28 deletion is complicated due to the dual regulation of the ubiquitination of FBXW7 itself and its substrates (Fig. 3). Complete knockdown of USP28 in mice triggers the auto-ubiquitination of FBXW7 and leads to substrate accumulation, while maintaining the single allele deletion of USP28 partially restores FBXW7 expression, and substrate protein levels are reduced in all mouse tissues [50]. USP28 deletion is associated with 53.2%-9.7% of breast cancer cases, which reveals that USP28 gene deletion-mediated disruption of FBXW7 auto-ubiquitination might correlate with breast cancer development in certain way, but this remains to be further investigated [51].

Overall, the regulation of FBXW7 is intertwined with a variety of positive and negative regulators and complex molecular pathways at the transcriptional, translational, and post-translational levels. An exploration of whether additional mechanisms regulate the intracellular expression of FBXW7 will help identify promising new breast cancer therapeutic targets.

## FBXW7 regulates breast cancer-related signaling pathways

### FBXW7 regulates NOTCH/NICD pathway

**Fig. 4:**
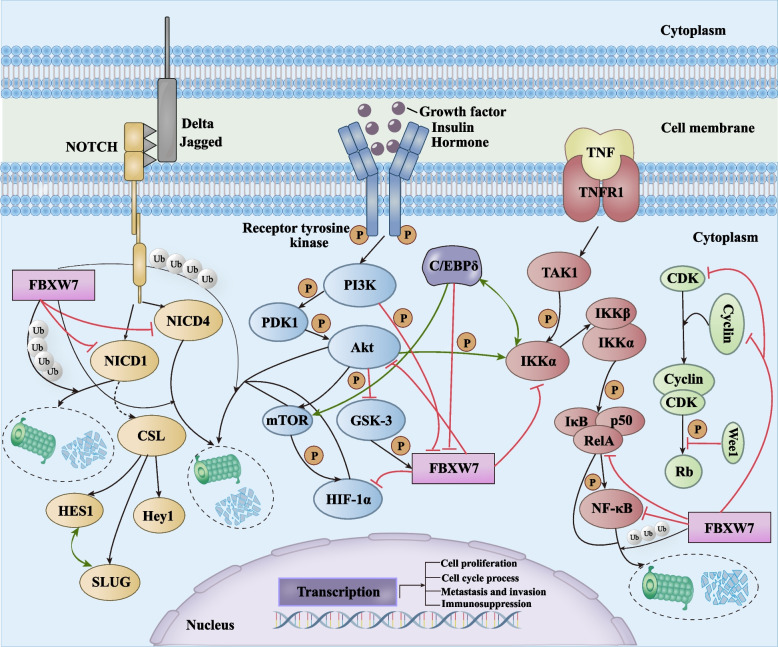
FBXW7 Regulates Breast Cancer-Related Signaling Pathways. Red arrows indicate inhibition, black arrows facilitation, and green arrows indicate interaction facilitation or crosstalk. Delta or Jagged binds to the NOTCH receptor to activate the NOTCH/NICD pathway. FBXW7 inhibits NICD1/NICD4 expression by promoting NICD1/NICD4 ubiquitination, which inhibits CSL DNA binding proteins downstream of the NOTCH pathway and target genes HES1, SLUG, and Hey1. Akt inhibits GSK-3-mediated upregulation of FBXW7, while FBXW7 in turn inhibits the PI3/Akt/mTOR pathway by promoting proteasomal degradation of Akt/mTOR/HIF-1α. C/EBPδ-mediated PI3K/Akt/mTOR-NF-κB crosstalk involves FBXW7. C/EBPδ promotes mTOR accumulation by inhibiting FBXW7 expression. IKKα is activated by Akt and C/EBPδ and inhibited as a substrate for FBXW7. CDK and cyclin create a complex that activates Rb when phosphorylated, while FBXW7 ubiquitinates and degrades CDK and cyclin to sustain the cell cycle. Uncontrolled transcription of downstream target genes by FBXW7 will cause anomalies in cell proliferation, cell cycle processes, metastasis, invasion, and immunosuppression. FBXW7, F-box and WD repeat domain-containing 7. HES1, HES family bHLH transcription factor 1. C/EBPδ, CCAAT/enhancer-binding protein-delta. CSL, CBFl/Suppressor of Hairless/Lag1. NICD1, NOTCH intracellular domain 1. mTOR, mechanistic target of rapamycin. NF-κB, nuclear factor-kappa B. HIF-1α, hypoxia inducible factor 1 alpha

The NOTCH gene encodes conserved cell membrane protein receptors. The binding of NOTCH ligands to the receptors triggers NOTCH activation and releases NOTCH protein fragments (NICD/ICN) via triple protein hydrolysis into the nucleus to activate transcription of downstream target genes, causing cancer cell processes [52]. It has been shown that FBXW7 is responsible for ubiquitin binding to NICD and promotes its proteasomal degradation, inhibiting NOTCH pathway activation [53]. Zhao et al*.* found that FBXW7 enhances the binding to NICD1 mediated by the melanoma-associated antigen A1 (MAGEA1) gene, which promotes the ubiquitination of FBXW7^SCF^-targeted NICD1, further reducing its binding to the transcriptional repressor CBFl/Suppressor of Hairless/Lag1 (CSL) in the nucleus, thus hindering MFC-7 and MDA-MB-231 cells proliferation [15]. FBXW7α specifically recognizes residues T2512/P2513 in the cdc4-phosphodegron of the nuclear molecules NICD1 and NICD4, while highly expressed prolyl-isomerase1 (Pin1) competitively binds to the site where NICD1/NICD4 docks with FBXW7α, disrupting the feed-forward molecular circuit between the three, inducing breast carcinogenesis and promoting breast cancer stem cell (BCSC) self-renewal [17]. These observations revealed that the relationship between FBXW7 and the NOTCH/NICD pathway is not a simple unidirectional one, but rather a cross-talk between upstream and downstream regulators of FBXW7 and NOTCH effector molecules (Fig. 4).

### FBXW7 regulates PI3K/Akt/mTOR pathway

High-frequency activation of the PI3K/Akt/mTOR pathway in BC greatly contributes to the transcription of downstream pro-oncogenic target genes such as HIF-1α, c-Myc and forkhead box O (FOXO), promoting cell proliferation, metastasis and drug resistance [54–56]. PI3K, the upstream signaling molecule of mTOR, regulates FBXW7 expression post-translationally through specific phosphorylation of the CPD motif. Aberrant stimulation of the signaling pathways PI3K/Akt and Wnt dramatically suppresses GSK-3β-mediated ubiquitination of FBXW7 [57]. C/EBPδ knock out (C/EBPδ-KO) breast tumor cells exhibit reduced Akt activity and decreased Ser9 phosphorylation of GSK-3β, which causes increased expression of FBXW7 downstream of GSK-3β. FBXW7 liberation from C/EBPδ transcriptional repression promotes polyubiquitination of endogenous mTOR and HIF-1α protein binding and inhibits intracellular accumulation, reducing lung metastasis in breast cancer model mice under hypoxic adaptation [16]. Furthermore, knocking down p50 and RelA, subunits of nuclear factor-kappa B (NF-κB), significantly attenuates IL-1-induced C/EBPδ expression [58]. Interestingly, while NF-κB interacts with and amplifies C/EBPδ in cancer cells, FBXW7, which is controlled by C/EBPδ, in turn triggers ubiquitination and destruction of NF-κB via GSK-3 phosphorylation, inhibiting the NF-κB cascade response in breast cancer [59]. Thus, the FBXW7 gene is a key hub of the C/EBPδ-mediated crosstalk between the Akt/mTOR/HIF-1α pathway and the NF-κB pathway, and its ubiquitination activity on different substrates deepens the linkage with the upstream and downstream signaling pathways (Fig. 4).

### FBXW7 regulates cyclin/CDK pathway

Cyclin/CDK forms a functional kinase complex at the GI/S to control the cell cycle into the S phase and aberrant activation of cyclin/CDK is linked to breast cancer carcinogenesis [60]. FBXW7 has been shown to interact with cyclin E, exploring the potential link between FBXW7 and the cyclin/CDK pathway would be useful in elucidating the mechanism of breast carcinogenesis [61]. Chromosomal instability (CIN) is a hallmark of cancer leading to cancer progression, tumor heterogeneity and drug resistance, and the deletion and mislocalization of the mitophagy-enriched envelope protein A (CENP-A) in breast cancer can cause abnormal chromosome division and rupture, promoting tumor progression and heterogeneity [62, 63]. Takada et al*.* detected less chromatin fraction CENP-A levels in FBXW7^−/−^ cells compared to FBXW7^+/+^ cells, rather than CENP-A for all cleavage products, and that ectopic introduction of FBXW7 into FBXW7^−/−^ cells significantly reduced cyclin E1 and CDK2 protein concentrations and rescued CENP-A expression [64]. The FBXW7-cyclin E/CDK2 targeting connection was also linked to cellular senescence phenotype [65]. Breast cancer development also involves FBXW7 control of cyclin D/CDK4/6. Sterol-regulatory-element binding protein 1 (SREBP1) is repressed by FBXW7 in a transcriptionally active version, and FBXW7 in turn indirectly represses SREBP1-dependent activation of cyclin D1 and downstream cyclin D1/CDK4/6 phosphorylation of Rb, blocking MCF-7 cell proliferation and causing partial G1 phase cell cycle arrest [66] (Fig. 4).

### FBXW7 regulates NF-κB pathway

NF-κB family proteins include p65 (RelA), RelB, c-Rel, p105/p50 (NF-κB1), and P100/52 (NF-κB2). High levels of NF-κB pathway accumulation are associated with an inflammatory, infiltrative clinicopathological breast cancer phenotype, and NF-κB nuclear accumulation is negatively correlated with ERα + expression in breast cancer [67, 68]. In TNBC, overexpression of the inhibitor of growth 5 (ING5) represses FBXW7, increasing p-NF-κB and activating the PI3K/Akt and NF-κB pathways, causing apoptosis, autophagy, migration, and invasion [69]. This indicates that in FBXW7-deficient breast cancers, the NF-κB signaling pathway that should be subject to normal E3 ubiquitin ligase binding and degradation is blocked, leading to increased NF-κB DNA binding activity and promoting tumor growth and metastasis. NF-κB, as a pro-inflammatory factor, also mediates the regulation of intrinsic and adaptive immune functions [70], and FBXW7 can regulate breast cancer through interaction with the pathway. Wu et al*.* found that silencing miR-182-5p in BT-549 and MDA-MB-231 cells significantly downregulates the expression of factors like p-p65, p-I-κB, tumor necrosis factor alpha (TNF-α), and IL-1β, while inhibiting FBXW7 restores NF-κB pathway-related complexes and promotes breast cancer apoptosis, proliferation, and decreased immune resistance [32] (Fig. 4).

Overall, the pleiotropic nature of FBXW7 as an E3 ubiquitin ligase receptor to recognize and bind substrates determines its fate in regulating multiple signaling pathways in breast cancer, and this process changes dynamically depending on the location of the FBXW7 substrate in the pathway and the biological function performed by the substrate.

## Connection between FBXW7 and breast cancer progression

Chromosome deletions, mutations, and methylation can cause FBXW7 to lose tumor suppressor function in breast cancer [71–73]. More importantly, by analyzing FBXW7 mRNA levels in 23 patients with recurrent breast cancer, the time from recurrence to death is significantly shorter in the FBXW7-Low group than in the FBXW7-High group, and the Ki67 labeling index (50.6%) and cyclin E staining positivity (24%) are significantly higher than in the FBXW7-High group (30.7%, 8.3%) [74]. Breast cancer patients with FBXW7 mutant somatic cells display a kinase signaling mutation profile and a negative prognosis for patients [75]. Furthermore, in 1900 breast cancer cases from publicly available datasets, FBXW7 mRNA levels do not segregate with ER status, with ER- patients exhibiting lower FBXW7 expression, disease-free survival (DFS) and overall survival (OS), and that in basal-like subtypes group with higher FBXW7 expression has higher DFS [76]. FBXW7 expression varies significantly between tumors, and low FBXW7 expression is associated with a poorer prognosis in breast cancer patients and could be a potential prognostic indicator for different breast cancer subtypes [72]. In summary, FBXW7 expression correlates with breast cancer development.

## Crosstalk between FBXW7 and binding proteins in BC progression

### Cell cycle process

**Fig. 5:**
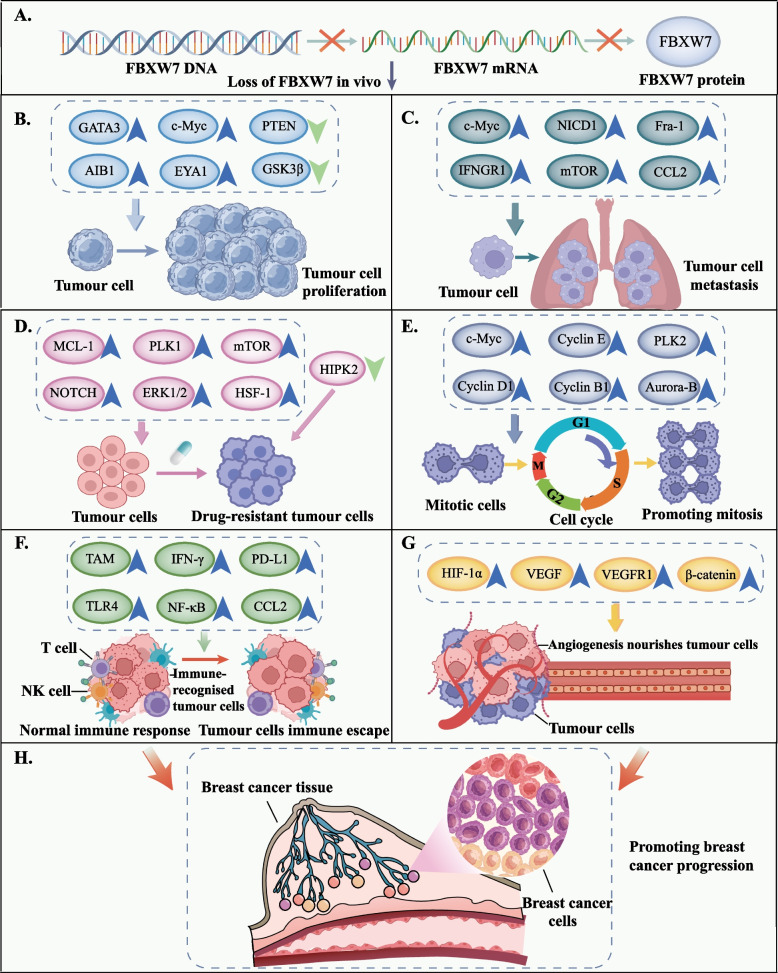
Loss of FBXW7 in vivo plays a role in breast cancer progression. Blue upward arrows indicate upregulation of the relevant target in breast cancer, and green downward arrows indicate downregulation. **A** Loss of FBXW7 in vivo. **B** FBXW7 deletion-mediated upregulation of GATA3, c-Myc, AIB1, and EYA1 and downregulation of PTEN and GSK3 promote breast cancer cell proliferation. **C** BXW7 deletion-mediated upregulation of c-Myc, NICD1, Fra-1, IFNGR1, mTOR, and CCL2 promotes breast cancer cell metastasis. **D** FBXW7 deletion-mediated upregulation of MCL-1, PLK1, ERK1/2, NOTCH, mTOR, and HSF-1 and downregulation of HIPK2 promote drug resistance in breast cancer. **E** FBXW7 deletion-mediated upregulation of c-Myc, PLK2, cyclin E, cyclin D1, cyclin B1, and Aurora-B promotes breast cancer cell cycle progression. **F** FBXW7 deletion-mediated upregulation of TAM, IFN-γ, PD-L1, TLR4, NF-κB, and CCL2 promotes the immune escape of breast cancer cells. **G** FBXW7 deletion-mediated upregulation of HIF-1α, VEGF, VEGFR1, and β-catenin promotes angiogenesis in breast cancer. **H** A series of malignant behaviors induced by the FBXW7 deletion promotes breast cancer progression. FBXW7, F-box and WD repeat domain-containing 7. GATA3, GATA-binding protein 3. AIB1, amplified in breast cancer 1. EYA1, eyes absent 1. PTEN, phosphatase and tensin homolog. GSK3, glycogen synthase kinase 3. NICD1, NOTCH intracellular domain 1. Fra-1, FOS-like 1 and AP-1 transcription factor subunit. IFNGR1, interferon gamma receptor 1. mTOR, mechanistic target of rapamycin. CCL2, C–C motif chemokine 2. MCL-1, myeloid cell leukemia-1. PLK1, polo-like kinase 1. ERK1/2, extracellular signal-regulated kinases 1 and 2. TAM, tumor associated macrophage. IFN-γ, interferon-gamma. PD-L1, programmed death ligand 1. TLR4, Toll-like receptor 4. NF-κB, nuclear factor-kappa B. HIF-1α, hypoxia inducible factor 1 alpha. VEGF, vascular endothelial growth factor. VEGFR1, vascular endothelial growth factor receptor 1

Through facilitating the ubiquitination and destruction of cell cycle-relevant molecules, FBXW7 has been implicated in the modulation of the cell cycle process in breast cancer [77] (Fig. 5E). Cyclin E and cyclin D1 are often amplified in breast cancer, leading to cell cycle dysregulation and uncontrolled cell proliferation [78]. Low FBXW7 expression in breast cancer deregulates cyclin E and D1, promoting aberrant cell division and G1/S phase transition [64, 66]. c-Myc is one of the classical targets of FBXW7, and genetic changes and accumulation of c-Myc in breast cancer severely affect disease recovery [79]. Eyes absent 1 (EYA1) is a class of oncogenes and is upregulated in breast cancer. Ubiquitination of FBXW7 targeting c-Myc can be rescued by EYA1 knockdown-mediated elevation of c-Myc pT58 and reduced endogenous c-Myc deposition, resulting in increased FBXW7-c-Myc binding, reduced c-Myc half-life and number of cycling cells [80]. Aside from the effects indicated above, FBXW7 downregulation also modulates the ubiquitination of related targets including cell cycle proteins G-associated kinase (GAK), p53, Aurora-B, γ-catenin, and CENP-A, which increases breast cancer cell cycle progression [21, 80–83]. Related studies on the regulation of the cell cycle by FBXW7 in breast cancer are summarized in Table 2.

**Table 2:** Roles of FBXW7 in the biological behavior of breast cancer

Upstream regulators	Expression in BC	Regulation of FBXW7	Target factor	Biological function	Ref
PLK2	Upregulation	Downregulation	↑Cyclin E	Promoting cell cycle progression	[44]
SREBP1	Upregulation	Downregulation	↑Cyclin D1; ↑CDK4/6; ↑Rb	Promoting cell cycle progression	[66]
Not applicable	Not applicable	Downregulation	↑Cyclin E1; ↑CDK2; ↓CENP-A	Promoting cell cycle progression	[64]
Not applicable	Not applicable	Downregulation	↑Aurora-B; ↑RepoMan	Promoting cell cycle progression	[82]
EYA1	Upregulation	Downregulation	↑c-Myc	Promoting cell cycle progression	[80]
PLK1	Upregulation	Downregulation	↑MCL-1	Promoting cell cycle progression	[84]
1,25 D	Downregulation	Downregulation	↑VDR; ↑c-Myc	Promoting cell cycle progression	[85]
FBXO45-MYCBP2	Upregulation	Downregulation	↑Cyclin B1	Promoting cell cycle progression	[86]
MiR-27a	Upregulation	Downregulation	↑Cyclin E; ↑EMT	Promoting cell cycle progression	[31, 61]
MAGEA1	Downregulation	Downregulation	↑NICD1	Promoting cell proliferation	[15]
Not applicable	Not applicable	Downregulation	↑MTDH	Promoting cell proliferation	[87]
GSK3β	Downregulation	Downregulation	↑GATA3	Promoting cell proliferation	[88]
PTEN	Downregulation	Downregulation	↑AIB1	Promoting cell proliferation	[89]
GSK3β	Downregulation	Downregulation	↑NONO	Promoting cell proliferation	[90]
SUMO chains	Upregulation	Downregulation	↑MCT4	Promoting cell proliferation	[91]
MiR-182-5p	Upregulation	Downregulation	↑TLR4; ↑NF-κB; ↑TNF-α; ↑IL-1β	Promoting cell proliferation	[32]
γ-tocotrienol	Downregulation	Downregulation	↑c-Myc	Promoting cell proliferation	[92]
USP36	Upregulation	Downregulation	↑c-Myc	Promoting cell proliferation	[93]
EglN2	Upregulation	Downregulation	Not applicable	Promoting cell proliferation	[94]
Wnt and PI3K/Akt signal	Upregulation	Downregulation	↑EYA1	Promoting cell proliferation	[57]
NOTCH/CCL2 axis	Upregulation	Downregulation	↑Mo-MDSCs; ↑TAMs	Promoting metastasis	[18]
C/EBPδ	Upregulation	Downregulation	↑HIF-1α; ↑IL-6Rα; ↑NICD1	Promoting metastasis	[24]
27-HC	Upregulation	Downregulation	↑LXR-α; ↑c-Myc; ↓SCP1; ↓PP2A	Promoting metastasis	[25]
NRG1-ERK1/2 axis	Upregulation	Downregulation	↑c-Myc; ↑Fra-1	Promoting metastasis	[47]
FAM83D	Upregulation	Downregulation	↑mTOR	Promoting metastasis	[95]
ELF5	Downregulation	Downregulation	↑IFNGR1; ↑IFN-γ; ↑PD-L1	Promoting metastasis	[96]
SGK3	Upregulation	Upregulation	↑INPP4B; ↑PIK3CA; ↓NDRG1	Promoting metastasis	[97]
ERα	Upregulation	Downregulation	↑C/EBPδ; ↓SNAI2	Inhibiting metastasis	[98]

↑, upregulation in BC. ↓, downregulation in BC. *BC* Breast cancer, *FBXW7* F-box and WD repeat domain-containing 7, *CDK4/6* Cyclin-dependent kinases 4 and 6, *Rb* Retinoblastoma, *EMT* Epithelial-mesenchymal transition, *CDK2* Cyclin-dependent kinases 2, *GATA3* GATA-binding protein 3, *AIB1* Amplified in breast cancer 1, *NICD1* NOTCH intracellular domain 1, *MCL-1* Myeloid cell leukemia-1, *NONO* One of the nuclear proteins, *TLR4* Toll-like receptor 4, *NF-κB* Nuclear factor-kappa B, *TNF-α* Tumor necrosis factor alpha, *MCT4* Monocarboxylate transporter 4, *Fra-1* FOS-like 1 and AP-1 transcription factor subunit, *IFNGR1* Interferon gamma receptor 1, *mTOR* Mechanistic target of rapamycin, IFN-γ Interferon-gamma, *PD-L1* Programmed death ligand 1, HIF-1α Hypoxia inducible factor 1 alpha, *C/EBPδ* CCAAT/enhancer-binding protein-delta, *INPP4B* Inositol polyphosphate 4-phosphatase type II, *PIK3CA* Phosphatidylinositol-4,5-bisphosphate 3-kinase catalytic subunit alpha. *NDRG1* N-Myc downstream regulated gene 1, *SCP1* Small C-terminal phosphatase 1, *PP2A* Protein phosphatase 2A, *SREBP* Sterol-regulatory-element binding protein 1, *1,25 D* 1,25 dihydroxy vitamin D, *PLK2* Polo-like kinase 2, *MAGEA1* Melanoma-associated antigen A1, *PTEN* Phosphatase and tensin homolog, *GSK3* Glycogen synthase kinase 3, *USP36* Ubiquitin-specific proteases 36, *EglN2* Egl-9 family hypoxia-inducible factor 2, *SUMO* SUMOylation, *NRG1* Neuregulin-1, *ERK1/2* Extracellular signal-regulated kinases 1 and 2, *ELF5* E74-like factor 5, *CCL2* C–C motif chemokine 2, ERα Estrogen receptor alpha, *FAM83D* Family with sequence similarity 83, member D, *SGK3* Glucocorticoid-regulated kinase 3, *27-HC* 27-hydroxycholesterol, *VDR* Vitamin D receptor, *RepoMan* Recruits PP1 onto mitotic chromatin at anaphase, *CIN* Chromosomal instability, LXR-α Liver X receptor-α, *CENP-A* Centromere protein A, *MTDH* Metadherin, *Mo-MDSCs* Monocytic myeloid-derived suppressor cells, *TAMs* Tumor associated macrophages. *SNAI2* slug, *MAGEA1* Melanoma-associated antigen A1, *EYA1* Eyes absent 1

### Proliferation

FBXW7 strictly controls the ubiquitination and degradation of proliferation-associated binding proteins, and its expression profile with substrates governs breast cancer cell proliferation and apoptosis [15, 87, 88] (Fig. 5B). In fact, FBXW7 binding to phosphatase and tensin homolog (PTEN) affects substrate ubiquitination. Amplified in breast cancer 1 (AIB1) is abundant in high-grade invasive ductal carcinoma, research has suggested that FBXW7α can bind to the C2 domain of PTEN and promote the ubiquitination of AIB1 bound to the PTEN phosphatase domain, reducing AIB1 transcriptional activity and inhibiting MCF-7 cell proliferation [89, 99]. Moreover, GSK3β-mediated CPD phosphorylation of FBXW7 is critical for breast cancer proliferation-associated substrate recruitment. NONO (one of the nuclear proteins) is an RNA-binding protein and silencing NONO significantly inhibits TNBC cell proliferation in vitro*/vivo* [100]. In the presence of GSK3β kinase initiation, FBXW7α recognizes the T428A/T432A phosphorylation site of NONO and promotes degradation of NONO-WT proteins, but not polyubiquitination of NONO mutant proteins [90]. Crosstalk between FBXW7 and substrates is regulated by various factors, but aberrant post-translational substrate changes can also change its binding status. Emerging evidence has suggested that breast cancer progression may be connected to SUMOylation's antagonistic effect on FBXW7-mediated monocarboxylate transporter 4 (MCT4) ubiquitination [91]. Related studies on the inhibition of proliferation by FBXW7 in breast cancer are summarized in Table 2.

### Metastasis and invasion

Metastatic breast cancer correlates with poor clinicopathological phenotype of invasion and drug resistance, downregulation of FBXW7 expression attenuates the ubiquitination and destruction of associated substrates, hence boosting breast cancer invasion and metastasis [18, 25, 95, 96] (Fig. 5C). HIF-1α and IL-6Rα overexpression in breast cancer patient sera creates a hypoxic environment and abnormal inflammation in tumor cells, promoting metastasis [101, 102]. C/EBPδ inhibits FBXW7 in ER + cells, releasing HIF-1α and IL-6Rα expression and activating downstream STAT3 phosphorylation, promoting in vitro calmodulin transformation, sphere formation, and patient-derived xenograft (PDX) tumor metastasis [24]. Breast cancer spread can also be driven by the EMT process, which makes epithelial cancer cells more metastatic and aggressive [103]. Anti-miR-223-3p upregulates FBXW7 translationally, which suppresses EMT, breast cancer cell migration, and invasiveness [33], but information on the specific targets of FBXW7 remains to be investigated. FBXW7 has a wide range of breast cancer targets, and imbalance in its binding to oncogenic substrates may drive tumor spread. For instance, cophosphorylation of Serum/glucocorticoid-regulated kinase 3 (SGK3) with GSK-3β enhances the binding of FBXW7 to metastasis suppressor N-Myc downstream regulated gene 1 (NDRG1) and promotes NDRG1's ubiquitination, increasing breast cancer invasion and metastasis [97]. This offers fresh perspectives on the application of FBXW7 as a therapeutic target for breast cancer. Related studies on the regulation of metastasis by FBXW7 in breast cancer are summarized in Table 2.

Taken together, various signaling pathways and gene crossing are involved in the process FBXW7 controls breast cancer proliferation, cell cycle, and metastasis. However, additional research into the mechanisms through which FBXW7 influences the biological behavior of breast cancer is still required, and exploring the suppressive effects of FBXW7 in depth might provide new targets for anti-breast cancer therapy.

### Angiogenesis

Advanced tumor staging, vascular invasion, and lymphatic metastasis have been linked to mutations in the FBWX7 gene [104] (Fig. 5G). The primary endothelial response that initiates angiogenic hypoxia signaling in breast cancer and particularly activates tumor-associated macrophages (TAMs) to produce pro-angiogenic cytokines and growth factors involves stable HIF-1α expression [105, 106]. FBXW7 indirectly regulates breast cancer angiogenesis through its interaction with HIF-1α, and high HIF-1α expression is frequently observed in FBXW7-deficient breast cancers along with angiogenesis and migration, suggesting that normal FBXW7-HIF-1α binding is essential for remodeling normal vascular structure and tumor microenvironment (TME) [107]. Similarly, modification of FBXW7-HIF-1α crosstalk by oncoproteins can disrupt FBXW7 to inhibit breast cancer angiogenesis, for example, TAR (HIV-1) RNA binding protein 2 (TARBP2) reduces the ubiquitination level and proteasomal degradation of HIF-1α by downregulating FBXW7-E3 ligase, which induces the formation of a hypoxic microenvironment and angiogenesis in breast cancer [108]. Furthermore, FBXW7 also regulates angiogenesis through direct or indirect control of vascular endothelial growth factor (VEGF) expression in tumors. VEGF-A promotes the release of VWF and osteoprotegerin from MDA-MB-231 and MCF-7 cell-induced endothelial cells (EC) and is pro-angiogenic [109]. Chiang et al*.* displayed that targeted binding of FBXW7 to miR-182 attenuates the proteasomal degradation of VEGF and HIF-1α, while reduced VEGF turnover promotes breast cancer angiogenesis and invasion [34].

It is meaningful to take into account the potential therapeutic strategies of reducing breast cancer angiogenesis by targeting FBXW7 and its downstream proteins in light of the critical function that FBXW7 plays in modulating angiogenesis.

### Immunosuppression

A major cause of recurrence, metastasis and drug resistance in breast cancers lacking effective anti-hormonal targets is the formation of immunosuppression in the tumor microenvironment, and clinical breast cancer treatment strategies have shown great interest in single agent co-immunosuppressive therapy [110]. Crosstalk between FBXW7 and related binding proteins regulates immune cell numbers, metastasis, and the premetastatic niche, playing a role in breast cancer immunosuppression [111, 112] (Fig. 5F). Interferon-gamma (IFN-γ) activates immune cells and induces differentiation and maturation, controlling a key aspect of the immunotherapeutic response. Breast cancer hijacks IFN-γ signaling and activates IFNGR and downstream cascade pathways, promoting inflammation and tumor spread [113]. In TNBC, ELF5 cannot bind to the enhancer region of FBXW7, which downregulates FBXW7 expression, reduces IFNGR1 ubiquitination, enhances IFN-γ signaling, increases immunosuppressed neutrophils, and promotes programmed death ligand 1 (PD-L1) expression [96]. This demonstrates that FBXW7 slows the advancement of breast cancer by abating the number of immune cells involved in immune evasion and present in TME. C–C motif chemokine 2 (CCL2) induces infiltration and accumulation of TAMs in breast cancer and is positively associated with cancer progression [114]. Yumimoto et al*.* uncovered that in breast cancer, FBXW7 deletion activates CCL2 promoter by reducing NOTCH turnover, which recruits monocytic myeloid-derived suppressor cells (Mo-MDSCs) and TAMs to the premetastatic niche and accelerates tumor metastasis and immunosuppression [115, 116]. In addition, FBXW7 has been shown to regulate solid tumor tolerance to immune checkpoint inhibitors (ICIs) and anti-PD-L1 therapy by blocking the nuclear factor of activated T cell 1 (NFAT1)/PD-L1 axis [117], which also offers new insights into the improvement of immunotherapeutic agents for breast cancer from the perspective of targeting FBXW7.

### Drug resistance

Multiple reports have confirmed that FBXW7 is closely linked to drug resistance in breast cancer [35, 118, 119] (Fig. 5D). Upregulation of FBXW7 has been found to feature prominently in promoting chemosensitization in breast cancer due to its part in the control of ubiquitination and proteasomal degradation of resistance-associated targets in tumors, such as Notch1-IC, myeloid cell leukemia-1 (MCL-1), and heat shock factor 1 (HSF-1). Increased Notch1-IC expression causes Adriamycin resistance in breast cancer cells, yet this process can be dramatically reversed by FBXW7 promoting proteasomal degradation of Notch1 [120]. HSF-1 is frequently overexpressed in drug-resistant cancer cells and promotes MDR formation through direct binding to the promoter of multidrug resistance 1 (MDR1). Activated ERK1/2 triggers FBXW7 down-regulation, which enhances HSF-1 binding to MDR1, leading to the emergence of paclitaxel-resistant breast cancer cells [121]. The anti-breast cancer mechanism of chemotherapeutic agents targeting microtubules, such as vincristine or paclitaxel, is to prolong or block mitosis, inducing apoptosis in mitotic cells [122]. Nevertheless, mitotic slippage (preventing premature cell exit from mitosis) promotes the production of mutant tetraploid cells, rendering tumor cells resistant to the drug. Compulsory expression of FBXW7 significantly downregulates the expression of MCL-1 and PLK1, and prevents cells from mitotic slippage and promoting apoptosis, hence restoring the sensitivity of MDA-MB-468 cells to paclitaxel [84].

It is possible that the role of FBXW7 is context-dependent and that its mediation of signaling cascade control extends beyond the reduction of tumor resistance. SRY-related HMG-box 4 (SOX4) binding to the FBXW7 promoter upregulates FBXW7 expression in ER + breast cancers, causing enhanced turnover of GATA-binding protein 3 (GATA3), and thus promoting tamoxifen tolerance [123]. This implies that FBXW7 mechanisms for controlling drug resistance in breast cancer are intricate. Therefore, a thorough investigation of the multiple mechanism underlying FBXW7 expression could benefit in the discovery of new chemotherapeutic approaches and possible indicators of drug resistance in breast cancer. Related studies on the regulation of drug resistance by FBXW7 in breast cancer are summarized in Table 3.

**Table 3:** Roles of FBXW7 in drug resistance of breast cancer

Drugs	Upstream regulators	Regulation of FBXW7 in BC	Target factor	Role	Ref
Not mentioned	↑Pin1	Downregulation	↑NOTCH1; ↑NOTCH4	Inducing resistance in BC	[17]
Adriamycin	↑MiR-188‑5p	Downregulation	↑c-Myc	Inducing resistance in BC	[35]
Nocodazole	Not applicable	Downregulation	↑MCL-1	Inducing resistance in BC	[119]
Adriamycin	↓HIPK2	Downregulation	↑Notch1-IC	Inducing resistance in BC	[120]
Taxol	↑ERK1/2	Downregulation	↑HSF-1; ↑MDR1	Inducing resistance in BC	[121]
Paclitaxel	Not applicable	Downregulation	↑MCL1; ↑PLK1	Inducing resistance in BC	[84]
Tamoxifen	↑SOX4	Upregulation	↓GATA3	Inducing resistance in BC	[123]
Vinca alkaloids	↑FBXO45-MYCBP2	Downregulation	↑Cyclin B1	Inducing resistance in BC	[86]
Rapamycin	Not applicable	Downregulation	↑mTOR	Raising sensitivity in BC	[124]
Epirubicin	↑NDR1	Downregulation	↑NICD; ↑NOTCH	Inducing resistance in BC	[125]
JQ1/I-BET	Not applicable	Downregulation	↑MCL-1	Inducing resistance in BC	[126]
Lobaplatin	Not applicable	Downregulation	↑MTDH	Inducing resistance in BC	[127]

↑, upregulation in BC. ↓, downregulation in BC. *BC* Breast cancer, *FBXW7* F-box and WD repeat domain-containing 7, *JQ1/I-BET* BET inhibitor JQ1. *ING5* Inhibitor of growth 5, *NDR1* Nuclear-Dbf2-related 1, *MTDH* Metadherin, *Pin1* Prolyl-isomerase1, *MCL-1* Myeloid cell leukemia-1, *PLK1* Polo-like kinase 1, *HSF-1* Heat shock factor 1, *MDR1* Multidrug resistance 1, *GATA3* GATA-binding protein 3, *mTOR* Mechanistic target of rapamycin, *NICD1* NOTCH intracellular domain 1, *SOX4*, SRY-related HMG-box 4, *ERK1/2* Extracellular signal-regulated kinases 1 and 2, *HIPK2* Homeodomain interacting protein kinase 2

## Targeting FBXW7 for breast cancer therapeutic strategies

As stated above, FBXW7 deletion reduces downstream target turnover and oncoprotein-dependent accumulation, which creates opportunities for targeted therapy of tumor cells. Here, we summarize the therapeutic strategy for targeting FBXW7 in breast cancer.

**Fig. 6:**
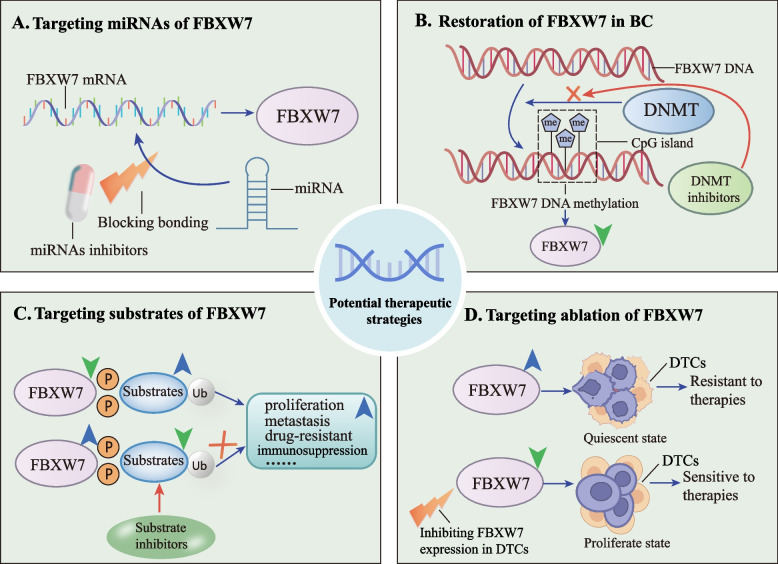
Targeting FBXW7 for breast cancer therapeutic strategies. Blue upward arrows indicate upregulation of the relevant target in breast cancer, and green downward arrows indicate downregulation. **A** Inhibition of miRNAs bound to FBXW7 mRNA restores FBXW7 expression in breast cancer. **B** FBXW7 gene methylation inhibition to restore FBXW7 expression in breast cancer. **C** Blocking breast cancer proliferation, metastasis, and drug resistance by inhibiting FBXW7 downstream substrates. **D** Promote the proliferation of dormant DTCs by inhibiting the expression of FBXW7 in DTCs, thereby suppressing drug resistance. BC, breast cancer. FBXW7, F-box and WD repeat domain-containing 7. MiRNA, microRNA. DNMT, DNA methyltransferase. DTCs, disseminated tumor cells

1) Targeting upstream miRNAs of FBXW7. The rapid spread of metastatic breast cancer and the lack of effective anti-hormonal targets necessitate the development of precisely targeted therapeutic techniques. mRNA regulators that target specific genes—miRNA mimics and antagonists—have shown positive effects in breast cancer treatment in combination with chemotherapy. Overexpression of miR-621 mimics downregulates F-box protein 11 (FBXO11), liberating p53 expression to promote apoptosis, and combining miR-621 mimics and paclitaxel plus carboplatin (PTX/CBP) increases cellular chemotherapy sensitivity and apoptosis [128]. The article's second point demonstrated that miRNAs exert an essential role in the advancement of breast cancer by binding to FBXW7's 3' UTR. MiR-27a antagonists upregulate FBXW7 and prevent invasion and metastasis in MDA-MB-231 and SKBR3 cells [31]. If the key issues of miRNA transport vectors and miRNA efficacy in cancer cells can be addressed in the future, mimics or antagonists targeting miRNAs upstream of FBXW7 may slow breast cancer progression. Natural items can block FBXW7 upstream miRNAs in addition to miRNA molecular agents. In invasive breast cancer, Honokiol downregulates miR-188-5p, which promotes the disruption of c-Myc by FBXW7 and reverses Adriamycin resistance [35] (Fig. 6A).

2) Restoration of FBXW7 tumor suppressor functions in breast cancer. Low FBXW7 expression is linked to breast cancer malignancy, therefore considering restoring its suppressive function and designing potential FBXW7 inducers may be a viable therapeutic strategy [72]. Promoter hypermethylation has been correlated to decreased FBXW7 gene transcription in primary breast cancer [73]. Recently, it has been explored that decitabine inhibition of DNA methyltransferase demethylates FBXW7, reducing MCL-1 expression and increasing cellular susceptibility to decitabine and Bcl-2 inhibitors [129]. Parallel to this, EZH2 inhibits FBXW7 and NUMB production by trimethylating histone H3 at Lys27 (H3K27me3), which activates anti-apoptotic genes and expresses T-cell multifunctional cytokines that attenuate immunosuppression in late tumor stages. FBXW7 demethylation reverses this [130]. This indicates that epigenetic targeted drugs may improve chemosensitivity in FBXW7-deficient breast tumors by acting as FBXW7 inducers. Low FBXW7 expression is also linked to overactive PKC and PLK1/2 signaling, which can be inhibited to slow its turnover [44, 45]. PKC inhibitor J-4 and PLK1/2 inhibitor onvansertib are in preclinical and clinical trials, respectively, and by inhibiting these molecules, endogenous FBXW7 expression may be restored and promote the turnover of breast cancer-associated oncogenic proteins [131, 132] (Fig. 6B).

3) Targeting downstream substrates of FBXW7. In FBXW7-negative breast tumors, due to the inability of FBXW7 to degrade typical drug-resistant proteins, drug sensitivity is often diminished. Inhibitors of downstream binding proteins may restore chemosensitivity. For instance, combining MCL-1 inhibitor sorafenib with nocodazole in MDA-MB-231 cells dramatically reverses drug resistance caused by low FBXW7 expression [119]. The mTOR inhibitor rapamycin significantly restores the poor FBXW7 expression caused by the family with sequence similarity 83, member D (FAM83D) and its pro-tumor effects [95]. Several AURKA inhibitors, an alternative target of FBXW7, have entered phase II/III clinical trials [133] (Fig. 6C). These findings demonstrate that FBXW7-negative breast cancer could be seen as an indicator of drug resistance and that inhibitors targeting substrates activated downstream of FBXW7 may relieve TNBC chemoresistance.

4) Inhibiting FBXW7 promotes sensitivity of BC latency DTCs. Tumor cells that have reached distant organs but have not yet formed clinically evident metastases are called disseminated tumor cells (DTCs). DTC in the bone marrow of breast cancer is in a quiescent single cell state and its low proliferation rate is a major cause of drug resistance [134]. FBXW7 degrades cyclin E to control cell cycle, yet this function precisely preserves DTCs in a quiescent state and high expression of the FBXW7 gene is detected in dormant cells [135]. FBXW7-ablated breast cancer cells break the stationary condition of DTCs and turn them into the drug-sensitive proliferative state. After treatment with FBXW7-ablation plus paclitaxel chemotherapy, DTCs become more sensitive to chemotherapy and fewer in number [136]. Thus, targeting FBXW7 ablation to stimulate DTCs from dormancy to proliferation may be a viable treatment to overcome breast cancer medication resistance (Fig. 6D).

## Conclusion

In summary, through regulating multiple intricate signaling pathways and targets, FBXW7 has a significant impact on the development of breast cancer (Fig. 5, Tables 2 and 3). The wide range of oncogenic targets allows FBXW7 to influence breast cancer growth in various ways and is of high value for targeted therapy. Moreover, detection of FBXW7 mutational status has potential as a prognostic marker for breast cancer and to be instrumental in establishing the appropriate, tailored treatment [18, 36]. To develop novel breast cancer therapies, future research may target FBXW7 mutations in vivo and downstream targets. Nevertheless, due to the intricate network of FBXW7, substrates, and regulators, its expression and function across cells remain uncertain. Significant challenges of FBXW7-targeted breast cancer therapeutic strategies include: how miRNA mimics or anti-miRNAs are transmitted is unknown; demethylation to restore FBXW7 expression may harm normally expressed genes; drugs cannot be targeted precisely due to the functional heterogeneity of FBXW7 isoforms; and the dual role of FBXW7 in breast cancer chemoresistance limits the action of chemotherapeutic agents. As a result, further technological advancements in the field of genomics and increased research on the complex role of FBXW7 in chemoresistance are required to target FBXW7 in breast cancer therapy. Further research on FBXW7's biological and molecular mechanisms in breast cancer will improve present therapy options and benefit more advanced breast cancer patients.

## Data Availability

Not applicable.

## Abbreviations

BC: Breast cancer
FBXW7: F-box and WD repeat domain-containing 7
mTOR: Mechanistic target of rapamycin
SCF: Skp1-Cullin1-F-box
RBX1: RING-box protein 1
CUL1: Cullin1
Skp1: S-phase kinase-associated protein 1
E1: Ubiquitin-activating enzymes
E2: Ubiquitin-conjugating enzymes
E3: Ubiquitin ligase
CPD: Cdc4 phospho-degron
GSK3β: Glycogen Synthase Kinase 3 Beta
KLF5: Kruppel-like factor 5
dsRNA: Double-stranded RNA
RANBP10: RAN binding protein 10
HES5: HES family bHLH transcription factor 5
C/EBPδ: CCAAT/enhancer-binding protein-delta
27-HC: 27-Hydroxycholesterol
p53BS: P53 binding site
TGIF1: TGF-β-induced factor 1
TGF-β: Transforming growth factor beta
NICD1: NOTCH intracellular domain 1
LncRNAs: Long non-coding RNAs
ceRNA: Competitive endogenous RNA
MiRNA: MicroRNA
EMT: Epithelial-mesenchymal transition
CircRNAs: Circular RNAs
USP28: Ubiquitin-specific proteases 28
TNBC: Triple-negative breast cancer
LSD1: Lysine-specific demethylase 1
ERK1/2: Extracellular signal-regulated kinases 1 and 2
PLK1/2: Polo-like kinase 1 and 2
PI3K: Phosphoinositide 3-kinase
SGK1: Serum- and glucocorticoid-inducible protein kinase 1
NRG1: Neuregulin-1
Fra-1: FOS-like 1 and AP-1 transcription factor subunit
CSN6: COP9 signalosome 6
SREBP1: Sterol-regulatory-element binding protein 1
Rb: Retinoblastoma
CENP-A: Centromere protein A
CIN: Chromosomal instability
GAK: G-associated kinase
PTEN: Phosphatase and tensin homolog
AIB1: Amplified in breast cancer 1
MCT4: Monocarboxylate transporter 4
ELF5: E74-like factor 5
HIF-1α: Hypoxia-inducible factor 1 alpha
BCSCs: Breast cancer stem cells
SGK3: Serum/glucocorticoid-regulated kinase 3
NDRG1: N-Myc downstream regulated gene 1
TAMs: Tumor-associated macrophages
VEGF: Vascular endothelial growth factor
TLR4: Toll-like receptor 4
NF-κB: Nuclear factor-kappa B
IFN-γ: Interferon-gamma
PD-L1: Programmed death ligand 1
TME: Tumor microenvironment
CCL2: C–C motif chemokine 2
Mo-MDSCs: Monocytic myeloid-derived suppressor cells
ICIs: Immune checkpoint inhibitors
NFAT1: Nuclear factor of activated T cell 1
MCL-1: Myeloid cell leukemia-1
HSF-1: Heat shock factor 1
MDR1: Multidrug resistance 1
GATA3: GATA-binding protein 3
FAM83D: Family with sequence similarity 83, member D
DTCs: Disseminated tumor cells
